# Rolling Circle Enhanced Detection of Specific Restriction Endonuclease Activities in Crude Cell Extracts

**DOI:** 10.3390/s22207763

**Published:** 2022-10-13

**Authors:** Kamilla Vandsø Petersen, Cinzia Tesauro, Marianne Smedegaard Hede, Camilla Pages, Lærke Bay Marcussen, Josephine Geertsen Keller, Magnus Bugge, Kasper Holm, Lotte Bjergbæk, Magnus Stougaard, Christian Wejse, Birgitta R. Knudsen

**Affiliations:** 1 Department of Clinical Medicine, Aarhus University, 8000 Aarhus, Denmark; 2 Department of Molecular Biology and Genetics, Aarhus University, 8000 Aarhus, Denmark; 3 VPCIR Biosciences Aps, 8000 Aarhus, Denmark; 4 Department of Pathology, Aarhus University Hospital, 8000 Aarhus, Denmark; 5 Department of Public Health, Aarhus University, 8000 Aarhus, Denmark

**Keywords:** restriction endonucleases, bacteria, rolling circle amplification, enzyme activity detection

## Abstract

Restriction endonucleases are expressed in all bacteria investigated so far and play an essential role for the bacterial defense against viral infections. Besides their important biological role, restriction endonucleases are of great use for different biotechnological purposes and are indispensable for many cloning and sequencing procedures. Methods for specific detection of restriction endonuclease activities can therefore find broad use for many purposes. In the current study, we demonstrate proof-of-concept for a new principle for the detection of restriction endonuclease activities. The method is based on rolling circle amplification of circular DNA products that can only be formed upon restriction digestion of specially designed DNA substrates. By combining the activity of the target restriction endonuclease with the highly specific Cre recombinase to generate DNA circles, we demonstrate specific detection of selected restriction endonuclease activities even in crude cell extracts. This is, to our knowledge, the first example of a sensor system that allows activity measurements of restriction endonucleases in crude samples. The presented sensor system may prove valuable for future characterization of bacteria species or strains based on their expression of restriction endonucleases as well as for quantification of restriction endonuclease activities directly in extracts from recombinant cells.

## 1. Introduction

Enzymes belonging to the family of restriction endonucleases (REs) are ubiquitously expressed in bacteria [1]. Their main function is to protect the host from viral infections by recognizing and cleaving viral DNA containing a specific consensus sequence, termed a restriction site [2]. To avoid digestion of host DNA, expression of most REs are coupled to a DNA methyltransferase that modifies and blocks potential restriction sites present in the host genome through the addition of methyl groups. Such modifications protect the endogenous restriction sites from RE cleavage [3]. REs are divided into different main types, designated type I, II, III and IV based on their structures, mode of DNA recognition, cleavage and required cofactors [4]. The type II enzymes, which are most commonly used for commercial or research purposes, are the best characterized REs. Members of this enzyme group typically cleave both DNA strands within or in close proximity to the recognition sequence [5]. Cleavage is accomplished with a classical type II nucleophilic substitution hydrolysis of the phosphodiester backbone in the target DNA, resulting in the generation of a 5′-phosphoryl and a 3′-hydroxyl end [6]. The resulting DNA breaks can have blunt ends or single-stranded overhangs often referred to as “sticky ends” due to their ability to anneal to complementary ends and facilitate ligation by, e.g., T4 DNA Ligase. Due to these specific characteristics, type II REs have been extensively used for many different applications including genetic engineering, DNA mapping and sequencing [7,8]. Besides their current scientific and commercial applications, REs may also be used to identify bacteria as they are differently expressed in different bacteria species or strains, as specified by the REs listed in the database, REBASE (http://rebase.neb.com, accessed on 20 August 2022) [9].

In line with the importance of REs, a number of different assays have been developed to measure their activities since their discovery in the 1960s [4]. Assays for the detection of RE activity are necessary, as REs are a fundamental part of the multiple tools used in biotechnology. Moreover, new REs are continuously being disovered and need to be characterized. Today, the most commonly used methods measure the ability of REs to cut fragments of DNA into pieces of specific sizes by using gel electrophoresis [10]. Filter assays, high performance liquid chromatography (HPLC) and enzyme-linked immunosorbent assays (ELISA) can also be used [11,12]. However, these methods are time-consuming, discontinuous and lack sensitivity. More recently, different real-time methods based on fluorescence resonance energy transfer (FRET) have been developed [13,14,15]. These techniques rely on short double-stranded DNA substrates or molecular beacons modified with a fluorophore and a quencher which are separated upon cleavage and allows fast and convenient measurement of RE activities [13,14,15]. However, these methods are not suitable for measurement of REs when present in crude samples where numerous unspecific endonucleases may cut the DNA substrates and separate the two components of the sensor system.

To allow for quantitative and gel-free measurements of specific RE activities in a manner that can be used even in crude extracts from, e.g., bacteria, we present a novel concept for RE activity detection. This concept is based on rolling circle amplification (RCA) of DNA circles generated specifically after RE processing of a hairpin-shaped DNA substrate. The assay setup resembles the previously published rolling circle enhanced enzyme activity detection (REEAD) [16] system for specific detection of eukaryotic or prokaryotic DNA topoisomerases and related enzymes [17,18,19,20], but distinguish itself by being the first of its kind that enable detection of a DNA cleaving enzyme without an intrinsic ligation activity. Detection is accomplished by using DNA substrates containing specific restriction sites with modified DNA ends that prevent ligation of undigested substrates, and a “helper” enzyme to facilitate ligation of RE digested products. Using this DNA substrate system, we demonstrate specific detection of RE activities even in crude cell extract. Moreover, the assay setup allowed us to distinguish two strains of *E. coli* based on their expression of REs, and can prove valuable for characterization and perhaps even discrimination of bacteria strains.

## 2. Materials and Methods

### 2.1. Reagents

All purified REs, CutSmart buffer, exonuclease I and III, T4 Polynucleotide Kinase and Quick-CIP Alkaline Phosphatase were purchased from New England Biolabs (Ipswich, MA, USA). All chemicals were purchased from Sigma–Aldrich (Søborg, Denmark).

### 2.2. DNA Substrates and Oligonucleotides

DNA was purchased from DNA Technology A/S (Aarhus, Denmark) or Microsynth AG (Balgach, Switzerland). RE sites are written in small letters.

- 5′-amine REEAD primer: 5′-/AmC6/CCAACCAACCAACCAAATAAGCGATCTTC

ACAGT-3′

- ID Detection probe: 5′-/FAM/CCTCAATGCACATGTTTGGCTCC-3′
- T4 EcoRI PA substrate: 5′-/AmC6/ATTCACTgaattcAGCGCTTAGGAGTGCATATACGATGCACTGTGAAGATCGCTTATGCATCGTATATGCACTCCTAAGCGCT

gaattcAGTGAAT/AmC6/-3′

- T4 EcoRI ID substrate: 5′-/AmC6/ATTTGACgaattcGTCGTATAGGAACTTCGAACGACTCGCCTCAATGCACATGTGGCTCCCGAGTCGTTCGAAGTTCCTATACGACgaattcGTCAAAT/AmC6/-3′
- T4 XhoI PA substrate: 5′-/AmC6/ATTCACTctcgagAGCGCTTAGGAGTGCATATACGATGCACTGTGAAGATCGCTTATGCATCGTATATGCACTCCTAAGCGCT

ctcgaAGTGAAT/AmC6/-3′

- T4 XhoI ID substrate: 5′-/AmC6/ATTTGACctcgagGTCGTATAGGAACTTCGAACGACTCGCCTCAATGCACATGTTTGGCTCC-CGAGTCGTTCGAAGTTCCTATAC

GACctcgagGTCAAAT/AmC6/-3′

- Cre EcoRI PA substrate: 5′-/AmC6/GCGACgaattcAATTATACGAAGTTATTCGCA

TACTGTGAAGATCGCTTATGAATAACTTCGTATAATTgaattcGTCGC/AmC6/-3′

- Cre XhoI PA substrate: 5′-/AmC6/GCGACctcgagAATTATACGAAGTTATTCGCA
- TACTGTGAAGATCGCTTATGAATAACTTCGTATAATTctcgagGTCGC/AmC6/-3′

### 2.3. Purification of T4 DNA Ligase 

The synthetic gene with a N-terminal 6 × His tag was cloned into the pQE-1 expression vector. The vector was transformed into competent *E. coli* BL21 cells (Promega) that were grown in 2 × TY medium supplemented with 25 µg/mL chloramphenicol. Expression was induced with 0.4 mM isopropyl b-D-1-thiogalactopyranoside (IPTG) at 28 °C at OD_600_ = 0.8. After 18 h the cells were harvested and resuspended in 50 mM NaPO_4_, pH 8.0, 0.1% Tween20, 10% glycerol and 20 mM imidazole. The cells were lysed by sonication on ice 6 × 30 s with 60 s break and interval set at 95% with a Branson Sonif 250, centrifuged and then filtered through a 0.45 µm filter (Sartorius Biotech). A final of 500 mM NaCl was added to the lysate which was then added to a pre-equilibrated Ni-NTA Sepharose column. The protein was eluted with 50 mM NaPO_4_ pH 8.0, 300 mM NaCl, 0.1% Tween20, 10% glycerol and 20 mM imidazole and dialyzed against 20 mM Tris-HCl pH 7, 40 mM NaCl, 1 mM DTT and 20% glycerol. Glycerol was added to a final of 50% and the ligase was stored at −20 °C.

### 2.4. Purification of Cre Recombinase 

The gene with a C-terminal 6 × His Tag was cloned into the pET28 expression vector. The vector was transformed into competent *E. coli* BL21 cells (Promega) that were grown in 2× TY with 50 μg/mL kanamycin. At OD_600_ = 0.6 protein expression was induced with 1mM IPTG and the cells were incubated 2.5 h at 37 °C with shaking. The cells were harvested and resuspended in 20 mL 50 mM NaPO_4_, pH 8.0, 0.1% Tween20, 10% v/v glycerol and 20 mM imidazole. Cells were kept on ice and 1:1000 saturated PMSF was added frequently. Cells were sonicated on ice 6 × 30 s with 60 s break and interval set at 95% with a Branson Sonif 250. NaCl was added to a final concentration of 500 mM and the sonicated cells were centrifuged. The cell lysate was filtered with a 0.45 μm filter (Sartorius Biotech) and loaded on a pre-equilibrated Ni-NTA Sepharose column. The column was washed with 300 mM NaCl, 50 mM NaPO_4_ pH 8, 20 mM imidazole, 10% v/v glycerol and the protein was eluted with 300 mM NaCl, 50 mM NaPO_4_ pH 8, 250 mM imidazole, 10% glycerol. The eluted fractions were dialyzed against 10 mM Tris-HCl pH 7, 300 mM NaCl, 1 mM DTT, 1 mM EDTA and 10% glycerol, flash frozen in liquid nitrogen and stored at −80 °C.

### 2.5. Preparation of Bacterial Lysates 

The two *E. coli* strains, RY13 and DH5α, were cultured in 2× TY medium supplemented with 100 µg/mL of ampicillin at 37 °C. At OD_600_ = 0.6 the bacteria were harvested by centrifugation at 4000× *g* for 10 min. The bacteria were resuspended in lysis buffer (10 mM Tris-HCl pH 7.5, 1 mM DTT and 1:1000 saturated PMSF), incubated 60 min on ice and vortexed 60 s with glass beads (150–212 µm). The lysate was spun 5 min at maximum speed and the supernatant used for the reaction. Colony forming units (CFU) of both strains were determined by streaking out a dilution series of 50 µL of the bacteria on a 2 × TY agar plate with 100 µg/mL of ampicillin. The resulting colonies were counted to determine CFU/µL.

### 2.6. Preparation of Functionalized Glass Slide

CodeLink HD slides (Surmodics, Eden Prairie, MN, USA) were divided into small 25 mm^2^ compartments using a mini pap pen (Thermo Fischer, Roskilde, Denmark). Then, 25 pmol of the 5′ amine REEAD primer was coupled to slides in print buffer (300 mM Na_3_PO_4_, pH 8). The slides were incubated in a humidity chamber with saturated NaCl overnight. The slides were then blocked in blocking buffer (50 mM Tris, 50 mM Tris-HCl, 50 mM ethanolamine, pH 9) for 30 min at 50 °C and subsequently washed in wash buffer 1 (4 × SSC, 0.1% SDS) for 30 min at 50 °C. 

### 2.7. T4 DNA Ligase Assisted RE Detection 

Ten pmol of both the T4 ID and PA DNA substrate (with RE site of interest) were incubated with 2 units purified RE or 20% reaction volume of lysate with appropriate CFU/µL in CutSmart buffer (50 mM Potassium Acetate, 20 mM Tris-acetate, 10 mM Magnesium Acetate, 100 µg/mL BSA pH 7.9) for 1 h at 37 °C. The RE reaction was heat-inactivated 10 min at 95 °C. Ligation was performed by the addition of 10 unit/μL of T4 DNA Ligase and 0.25 mM ATP for 60 min at 16 °C. Ligation was inactivated 10 min at 95 °C. To remove non-circular products the samples were incubated with 2 units of both exonuclease I and III for 1 h at 37 °C. Exonuclease digestion was inactivated 10 min at 95 °C. The resulting DNA circles were hybridized to the functionalized glass slides 1 h in a humidity chamber at 37 °C. The slides were washed 1 min in wash buffer 2 (100 mM Tris-HCl pH 7.5, 150 mM NaCl, and 0.3% SDS), 1 min in wash buffer 3 (100 mM Tris-HCl pH 7.5, 150 mM NaCl, and 0.05% Tween20), and dehydrated 1 min in 96% ethanol. RCA was performed 60 min at 37 °C with 1 unit/μL Phi29 DNA Polymerase (VPCIR Bioscience ApS, Denmark) in 50 mM Tris-HCl, 10 mM MgCl_2_, 10 mM (NH_4_)_2_S0_4_, 4 mM DTT pH 7.5, 0.2 μg/μL BSA and 250 μM dNTPs in a humidity chamber. The reaction was stopped by washing in wash buffer 2, 3 and 96% ethanol. Two pmol of the FAM ID detection probe was added in 20% formamide, 300 mM NaCl, 30 mM sodium citrate and 5% glycerol and the slide was incubated 30 min at 37 °C in a humidity chamber. The slide was washed 10 min in wash buffer 2, 5 min in wash buffer 3 and 1 min in 96% ethanol. Vectashield (Vector laboratories, Burlingame, CA, USA) and cover glass (Hounisen, Skanderborg, Denmark) were added and the slide were analyzed with a fluorescent microscope (Olympus IX73) at 60× magnification with a GFP filter. Ten pictures were acquired per sample and the number of signals per image frame were analyzed using the open source ImageJ Fiji software (version: 2.0.0-rc-69/1.52p/ Java 1.8.0_202 (64-bit)). 

### 2.8. Cre Assisted RE Detection 

Ten pmol of the Cre PA substrate (with RE site of interest) was incubated with 2 units purified RE or 20% reaction volume of lysate with appropriate CFU/µL in CutSmart buffer and 1 unit/µL AP for 1 h at 37 °C. Then, 50 mM EDTA and 1 unit/µL Cre recombinase was added and the circularization was performed 1 h at 37 °C and inactivated by 10 min incubation at 95 °C. The DNA circles were hybridized to the functionalized glass slides 1 h in a humidity chamber at 37 °C. The slides were washed 1 min in wash buffer 2, 1 min in wash buffer 3, and dehydrated 1 min in 96% ethanol. RCA was performed 2 h at 37 °C with 1 unit/μL Phi29 DNA polymerase in 50 mM Tris-HCl, 10 mM MgCl2, 10 mM (NH4)2S04, 4 mM DTT pH 7.5, 0.2 μg/μL BSA, 250 μM dNTPs and 12.5 μM atto-488-dUTP (Jena Bioscience, Germany) in a humidity chamber. The reaction was stopped by washing 10 min in wash buffer 2, 5 min in wash buffer 3 and 1 min 96% ethanol. Vectashield and cover glass were added and the slide were analyzed with a fluorescent microscope (Olympus IX73) at 60× magnification. Ten pictures were acquired per sample and the number of signals were analyzed using the ImageJ software.

### 2.9. Statistics 

Data were analyzed using the GraphPad Prism software and plotted as mean with standard deviation. Unpaired *t*-tests were used as indicated in the figure legends.

## 3. Results and Discussion

### 3.1. T4 DNA Ligase Assisted Restriction Endonuclease Detection

**Figure 1:**
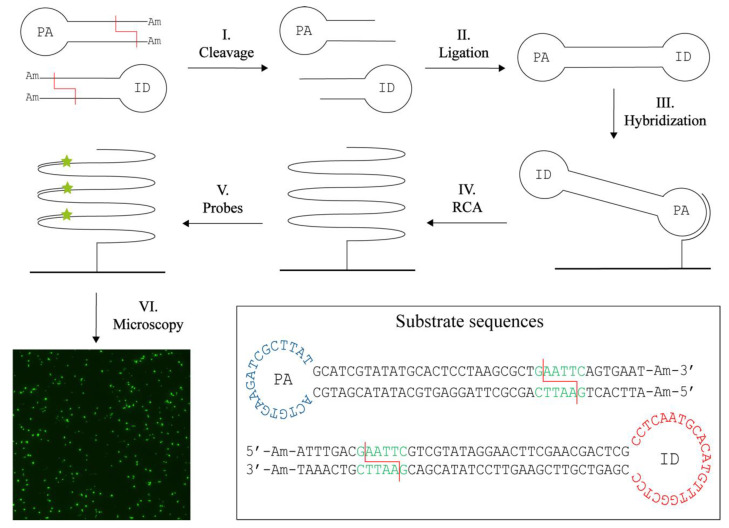
T4 DNA ligase assisted restriction endonuclease (RE) detection. The method uses two DNA hairpin substrates containing a specific restriction site (red line) and amine (Am) blocked ends. The loop of one substrate contains a primer annealing (PA) sequence, while the other contains an identification (ID) sequence. Upon cleavage (I) the blocked ends are removed and the two substrates can hybridize and be ligated with the T4 DNA Ligase (II). Note that only products containing both the PA and ID sequence will be detected. The circular product is hybridized to a surface anchored oligonucleotide (III) that also function as a primer for rolling circle amplification (RCA) (IV). This generates a long tandem repeat product to which fluorescently labelled probes can be annealed (V). The resulting signals can be visualized in a fluorescence microscope (VI). One signal corresponds to two RE digestions and hence provide a quantitative measure. The sequences and folding of the DNA substrates are found in the box with the restriction site (green), here exampled with the restriction site for EcoRI, the cleavage sites (red lines), the PA sequence (blue), the ID sequence (red) and the ends blocked with amines (Am).

The principle behind the novel concept for RE activity detection, termed T4 DNA Ligase assisted RE detection, is schematically illustrated in Figure 1. The method relies on two different hairpin-shaped DNA substrates. Each substrate is composed of a double-stranded stem region with a specific restriction site and ends modified with 3′- and 5′-amine groups to prevent ligation of uncut substrates. The single stranded loop region in one of the substrates contains a primer annealing (PA) sequence, while the other substrate contains an identification (ID) sequence. Upon cleavage by the target RE (step I), the modified DNA ends are removed and the cleaved DNA hairpins can be ligated pairwise by T4 DNA Ligase (step II). Ligation generates a circular dumbbell-shaped product with two loops containing the PA sequence and the ID sequence. The combination of the PA and the ID sequences in one circular DNA molecule ensures detection. The PA sequence facilitates annealing to a surface-coupled oligonucleotide (step III). The surface is blocked beforehand with amines to avoid unspecific binding of DNA. The surface-coupled oligonucleotide also functions as a primer that supports RCA by Phi29 DNA Polymerase (step IV). RCA results in the generation of an approximately 10^3^ tandem repeat product with a sequence complementary to the circular product. This product can subsequently be detected at the single molecule level in a fluorescence microscope upon hybridization of fluorescent probes complimentary to the ID sequence (step V and VI). In this manner, only products composed of both substrates are detected. Even though signals will be lost if two substrates of the same type are combined, we chose to use two different hairpin DNA substrates in an attempt to increase specificity of the assay and minimize the number of false positive signals. These may be generated upon unspecific closure of one substrate if the amine modifications were removed by, e.g., exonuclease activities in crude samples. Since the sensor system relies on isothermal amplification of circularized products alone, it was anticipated that the assay allows for quantitative detection of RE activity. 

### 3.2. Specific Detection of Purified RE Activities

**Figure 2:**
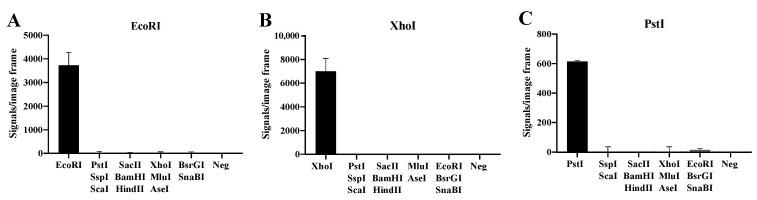
Specific detection of purified REs. The DNA substrate systems for EcoRI (**A**), XhoI (**B**) and PstI (**C**) were incubated with their target RE and a mixture of two to three non-target REs (SacII, SspI, BamHI, HindIII, MluI, AseI, SnaBI and BsrGI. A negative control (Neg) with buffer alone was included. The data are plotted as the mean of signals/image frame with standard deviation, *n* = 3.

To address the specificity of the basic substrate system illustrated in Figure 1 towards selected REs, three substrate pairs with RE sites recognized by EcoRI, XhoI and PstI were designed. Each of the substrate pairs were incubated with two units of their target enzyme or a mixture of tw units each of two to three non-target REs including EcoRI, XhoI, EcoRI, PstI, SacII, SspI, BamHI, HindIII, MluI, AseI, SnaBI and BsrGI (Figure 2). Following digestion, the REs were heat-denatured and the digested substrate pairs ligated by T4 DNA Ligase. The resulting circles were subjected to RCA and detected by hybridization with fluorescent probes. The florescent RCA products were visualized in a fluorescent microscope and the number of signals generated in each sample were counted. As evident, each substrate pair allowed detection of the target RE only (EcoRI in Figure 2A, XhoI in Figure 2B, and PstI in Figure 2C), while addition of the indicated mixtures of non-target enzymes gave no or close to no background signal. Note, that the lack of signals observed in the absence of the target RE was not due to buffer components present in one or more of the non-target RE preparations, since addition of the target RE could restore normal signal levels (data not shown). 

These results demonstrate that the RCA-based sensor system is specific for the target RE and do not react with non-target REs when analyzing purified enzymes. Moreover, the sensor system was able to measure down to 0.1–0.2 units of purified target REs (see Supplementary Figure S1). This suggests that the presented RCA-based sensor system may be a convenient new assay for assessment of RE activities in a gel-free system.

### 3.3. Detection of REs in Cell Extracts

**Figure 3:**
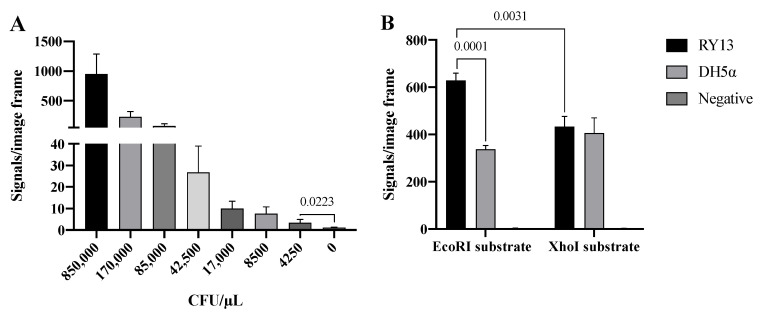
Detection of RE activity in cell extracts. (**A**) Titration of 850,000–4250 CFU/μL RY13 *E. coli* with the EcoRI substrate system. (**B**) Extract from 170,000 CFU/μL RY13 and DH5α on EcoRI and XhoI substrates. The results are plotted as the mean of signals/image frame with standard deviation, *n* = 3. *p*-values indicating significant difference as calculated with an unpaired *t*-test are marked in the graphs.

As already mentioned, REs are differently expressed in bacterial species as part of their anti-virus protective system. Bacteria express different combinations of REs and it may hence be possible to distinguish bacteria strains or species based on their expression of RE activities [21,22]. To address if the developed RCA-based RE sensor system can be used to detect specific RE activities present in crude extracts from bacteria, we took advantage of two *E. coli* strains: RY13 and DH5α. RY13 naturally overexpresses EcoRI and was originally used to purify EcoRI [23], while DH5α does not express EcoRI according to the REBASE database. Initially, the ability of the EcoRI specific sensor system to detect EcoRI in extracts in a decreasing number of RY13, ranging from 850,000–4250 CFU/μL, was measured. The results are graphically depicted in Figure 3A and indicate a quantitative detection of RY13 extracts with a significant difference between 0 and 4250 CFU/μL in the utilized experimental setup. 

The specificity of the RCA sensor system was addressed by comparing the number of signals obtained with extracts from RY13 or DH5α cells using substrate pairs with EcoRI or XhoI recognition sites. As mentioned above, RY13 and not DH5α expresses EcoRI, while none of the strains express XhoI according to REBASE. In the experimental setup, extracts from 170,000 CFU/μL, which is well above the detection limit according to Figure 3A, was prepared and analyzed in the RCA sensor system using each of the two substrate pairs. The number of signals were counted and the results are graphically depicted in Figure 3B. When used for analysis of cell extracts, the assay setup was associated with extensive background noise as evident from the number of signals observed after incubation with either of the cell extracts with the substrate pair containing the XhoI restriction site, or the DH5α cell extract with the substrate pair containing an EcoRI site. Consistent with the expected EcoRI activity in RY13, extracts from RY13 resulted in a significantly higher number of signals than DH5α when using the substrate pair containing the EcoRI site. However, the difference was only two-fold and the number of unspecific signals observed in DH5α on both of the DNA substrates were unacceptably high for the sensor system having any practical use for analysis of REs in cell extracts. 

As schematically illustrated in Figure 1, the substrate pairs were chemically modified with amine groups in both the 3′and 5′ ends to prevent ligation of undigested DNA substrates, and ensure that ligation could only proceed upon removal of the ends by RE digestion. It is possible, however, that the high level of background signals observed in Figure 3B was caused by the DNA substrates being cleaved by unidentified REs present in the cell extracts. Such enzymes could generate sticky ends and facilitate the generation of circles that would be amplified to generate detectable products. Unspecific endo- or exonuclease activities could potentially also remove the terminal amines and create DNA ends, which might be joined by T4 DNA Ligase even in the absence of sticky ends. The T4 DNA Ligase can to some extent facilitate ligation of more or less random DNA ends as long as they contain a 5′-phosphate and a 3′-hydroxyl end [24,25]. To identify the potential reasons behind the high level of background signals, the circular DNA products generated by cell extracts were PCR amplified and sequenced (see Supplementary Figure S2). The result of this analysis revealed that approximately 50% of the circles were generated by joining the two substrates at the EcoRI site, while the other 50% of the circles represented a large variety of products joined at multiple different sites in the two substrate stems. This is consistent with the observed level of background signals and suggests that the two substrates can be processed by unspecific endo- or exonucleases present in cell extracts to generate products which can be joined by the T4 DNA Ligase and result in unspecific signals. 

### 3.4. Specific End-Joining by Cre Recombinase 

The sequence analysis of DNA circles generated in crude cell extracts suggested that the high level of background observed when analyzing RE activities (Figure 3B) was due to unspecific ligation of DNA products generated by various nuclease activities present in cell extracts. Several attempts to decrease this background by using high fidelity ligases including T7 DNA Ligase and Pfu DNA Ligase failed (data not shown). A different strategy was therefore attempted, taking advantage of the highly sequence-specific cleavage/ligation activity of Cre recombinase to generate circles after RE digestion. Cre belongs to the family of tyrosine recombinases, which are characterized by mediating conservative site-specific recombination. Cre recognizes and cleaves DNA in a highly sequence specific manner, creating an intermediate 3′-phosphotyrosyl linkage and a free 5′-hydroxyl end, which is used as a nucleophile in the subsequent ligation across DNA molecules that are recombined. Cre catalyzes site-specific and conservative recombination between specific sequences termed LoxP sites, which are comprised of two 13 bp inverted repeats (ATAACTTCGTATA) separated by an 8 bp spacer. Cre specifically recognizes the inverted repeats and cleave one base within the 8 bp spacer region generating a covalent 3′-linkage with the DNA and a protruding six base 5′-hydroxyl end [26,27]. Hence, in contrast to the T4 DNA ligase and related enzymes, Cre reacts in a very specific and strictly sequence-dependent manner. This reaction scheme has previously allowed for detection of Cre activity using DNA substrates with a so-called half LoxP site containing a single inverted repeat and a half spacer region. Such substrates has allowed for investigations of the separate cleavage-ligations steps of Cre catalysis [28] as well as for detection of Cre activity in a REEAD-based setup [29].

**Figure 4:**
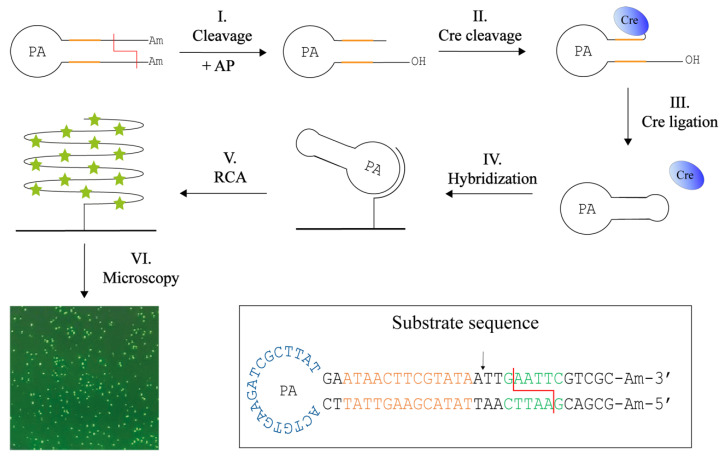
Cre-assisted RE detection. This method uses one DNA hairpin substrate containing Cre (orange) and RE (red) recognition sites in the stem, the primer annealing (PA) sequence in the loop and amine (Am) blocked ends. Following digestion in the presence of alkaline phosphatase (AP) that removes the 5′ phosphate end (I), Cre will bind and cleave at the 3′ end of the DNA substrate, leaving Cre covalently attached (II) and leave the substrate with a 5′ overhang of six bases. Cre is able circulariza the substrate by ligation of the protruding 5′ end (III). This circle is hybridized to a primer immobilized on a microscopic glass surface (IV). From the primer, rolling circle amplification (RCA) is also initiated. RCA is performed with ATTO-488 labelled dUTPs which are incorporated into the long tandem repeat product (V). This enables visualization in a fluorescent microscope (VI). The sequence and folding of the DNA substrate can be seen in the box with the Cre recognition site (orange) and Cre cleavage site (arrow), RE site (green), here examples with EcoRI, and RE cleavage (red line), the PA (blue) in the loop, and the amines (Am) blocking the ends.

We therefore envisioned that upon insertion of the Cre recognition sequence (ATAACTTCGTATA) in the RE specific substrates, Cre recombinase may allow for specific closure of RE generated products, at least for REs that generate 5′ overhangs of suitable length. This is the case for the two REs, EcoRI and XhoI, tested so far. Based on the requirements for Cre cleavage and ligation, two substrates for the detection of EcoRI or XhoI was designed. The method termed Cre-assisted RE detection is illustrated in Figure 4. This setup is based on a hairpin-shaped DNA substrate that contains the specific Cre and RE recognition sites in the stem, amine-functionalized 3′ and 5′ ends (to prevent circularization of unprocessed substrates), and a PA site in the loop. Cleavage by the target RE (EcoRI or XhoI) removes the amine-functionalized ends and leaves a six-base protruding 5′-phosphate end and a recessed 3′-hydroxyl end three bases downstream from the Cre cleavage site (step I). Since Cre recombinase requires a 5′-hydroxyl, alkaline phosphatase (AP) is added during the cleavage reaction (step I). The resulting product can be cleaved by Cre-recombinase which forms a covalent cleavage intermediate (step II). Cre can subsequently circularize the substrate by ligating the protruding 5′-end (note that like other members of the tyrosine recombinase family, Cre ligates 5′-hydroxyl ends only) (step III). As in the previous method, the DNA circles were hybridized to a surface-anchored primer (step IV), from which RCA also can be initiated. In this setup, RCA was performed with the incorporation of fluorescent nucleotides (V). The resulting fluorescent signals were detected using a fluorescence microscope and counted as the previous described method. Since Cre is highly selective with regard to both DNA sequence and structure, and since a Cre cleavable substrate is only formed upon reaction with the specific target RE, we anticipated that this substrate design could allow for specific detection of the selected REs even when present in crude extracts. Additionally, it was anticipated that due to the high specificity of the Cre reaction, the use of sequence specific probes for detection would be unnecessary when using this design. 

### 3.5. Detection of REs with Cre-Assisted RE Detection

**Figure 5:**
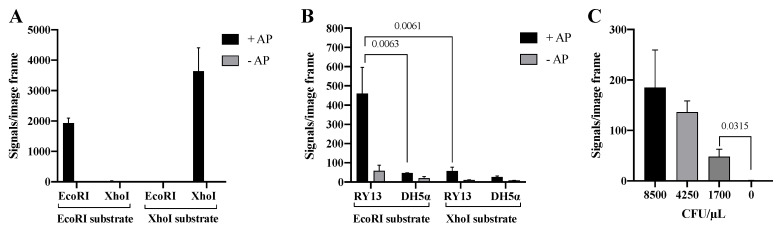
Cre assisted RE detection. (**A**) Purified EcoRI and XhoI on EcoRI and XhoI DNA substrates with alkaline phosphatase (+AP, black bar) and without alkaline phosphatase (−AP, gray bar) to remove the 5′-phosphate group before Cre assisted circularization of the substrate. (**B**) Extract from RY13 and DH5α on both EcoRI and XhoI DNA substrates with AP (+AP, black bar) or without AP (−AP, gray bar). (**C**) Titration of 8500–1700 CFU/μL RY13 *E. coli* on the EcoRI DNA substrate. All data are plotted as mean of signals/image frame with standard deviation, *n* = 3. *p*-values indicating significant difference as calculated with an unpaired t-test are marked in the graphs.

The Cre assisted RE detection strategy was tested by incubating two units of purified EcoRI or XhoI with DNA substrates containing restriction sites specific for each of the enzymes. The involvement of Cre in the circularization of RE products was verified by testing the effect of AP catalyzed dephosphorylation of the 5′-phosphate ends generated by RE cleavage (recall that Cre ligation requires a 5′-hydroxyl end). As evident from the graphical depiction of the results (Figure 5A), signals were only obtained when either of the two REs were incubated with the substrate containing the matching RE recognition sequence. Moreover, the generation of signals depended on 5′ dephosphorylation by AP, which is consistent with Cre mediated end-joining. 

To investigate if the Cre mediated end-joining allowed for specific detection of RE activities in crude cell extracts, the Cre assisted RE detection strategy was tested using extracts generated from 170,000 CFU/μL of RY13 or DH5α cells as described above. The results graphed in Figure 5B demonstrates generation of a high number of signals only when extract from RY13 was incubated with the EcoRI specific substrate. Incubation of the RY13 extract with the XhoI specific substrate or DH5α extract with either of the substrates resulted in only trace amounts of signals. This is consistent with the lack of XhoI expression in RY13 and the lack of both EcoRI and XhoI expression in DH5α. Hence, replacing T4 DNA Ligase with the far more specific enzyme Cre recombinase to facilitate end-joining of RE products reduced the amount of background signals considerably and allows for detection of target REs even in crude cell extracts. In agreement with Cre recombinase being responsible for the circularization, generation of signals was greatly inhibited when AP catalyzed 5′-dephosphorylation was omitted (see gray bars in Figure 5A,B). The results with the Cre assisted RE detection strategy indicates that the assay provides a quantitative analysis of extracts from a serial dilution of RY13 cells (see Figure 5C) and is able to detect 1700 CFU/μL in the utilized experimental setup.

## 4. Conclusions

Since their discovery more than half a century ago, REs have been highly valuable tools in biotechnological research and industry. In particular, they have been used for countless cloning and sequencing procedures. Without a comprehensive knowledge of these enzymes it is difficult to imagine the biotechnological progress we take for granted today. Nevertheless, standard measurements of RE activities still rely on relatively tedious processes that requires access to specialized gel-electrophoretic equipment. Although FRET-based alternatives which rely on the separation of fluorophore-quencher pairs upon RE reaction have been presented, these are all sensitive to the presence of even trace amount of contaminating endonucleases in a given sample. In the present study, we demonstrate proof-of-concept of a novel RCA-based system for detection of RE activities. This system is based on amplification of specific DNA circles generated upon ligation of RE digested hairpin-shaped DNA substrates. The initial detection system relied on ligation of RE digested products by T4 DNA Ligase. This system allowed for specific detection of purified REs, but resulted in an unacceptable high background level when used for detection of REs in crude cell extracts. Sequencing of the generated DNA circles demonstrated that the large number of background signals were caused by unspecific ligation of a variety of products resulting from unspecific digestion of the substrates. This could not be prevented even by replacing T4 DNA Ligase with a high-fidelity ligase and rendered the system unsuited for measurements in crude biological samples.

A different setup taking advantage of the DNA end-joining capacity of the highly sequence specific Cre recombinase was therefore attempted. In this setup, the RE specific DNA hairpin substrate were designed in such a manner that Cre mediated DNA end-joining only occurred upon RE digestion by the correct RE. This setup proved to be highly specific even in crude cell extracts. To our knowledge, this is the first time a detection system has been presented that allow for measurement of RE activities in crude samples. Since available evidence suggests that different bacteria species or strains may be characterized by their expression of RE activities, the presented system may prove valuable for characterization and maybe even discrimination of bacteria strains in the future. Additionally, the system can allow for quantification of RE activities directly in extracts from recombinant cells used for biotechnological or research purposes. Moreover, the method can be coupled to a simple colorimetric or chemiluminescent readout as described by Keller et al. [30]. Due to the substrate requirements of the highly specific Cre recombinase the presented system is restricted to the detection of RE creating a 5′-overhang. However, by the use of other highly specific recombinases/resolvases capable of ligating 3′-overhangs such as the HIN/GIN resolvases [31], substrate systems for detecting of other types of REs can be envisioned in the future. 

## 5. Patents

The authors C.T., M.S. and B.R.K. declare that they are named inventors on the pending unpublished patent application PCT/EP2022/057172 relating to the technology described in here, filed in the name of VPCIR Biosciences ApS.

## Data Availability

Fastq files from the supplementary data are available upon request from the authors.

## Supplementary Materials

The following supporting information can be downloaded at: https://www.mdpi.com/article/10.3390/s22207763/s1, Figure S1: Detection of purified REs; Figure S2: Sequencing of DNA circles.
