# Supplementary material for: Rolling Circle Enhanced Detection of Specific Restriction Endonuclease Activities in Crude Cell Extracts
Source: Sensors (Basel). 2022 Oct 13;22(20):7763. doi: 10.3390/s22207763 (PMC9608553; doi:10.3390/s22207763)
Supplement: Supplementary file 1 [file sensors-22-07763-s001.zip › sensors-1909946-supplementary.pdf]

sensors

Supplementary Materials: Rolling Circle Enhanced Detection of Specific Restriction Endonuclease Activities in Crude Cell Extracts

Supplementary S1

Detection of purified REs

The ability of the T4 DNA ligase assisted RE detection method to quantitatively detect RE activity was investigated using a titration of purified EcoRI and XhoI on their respective target DNA substrate.

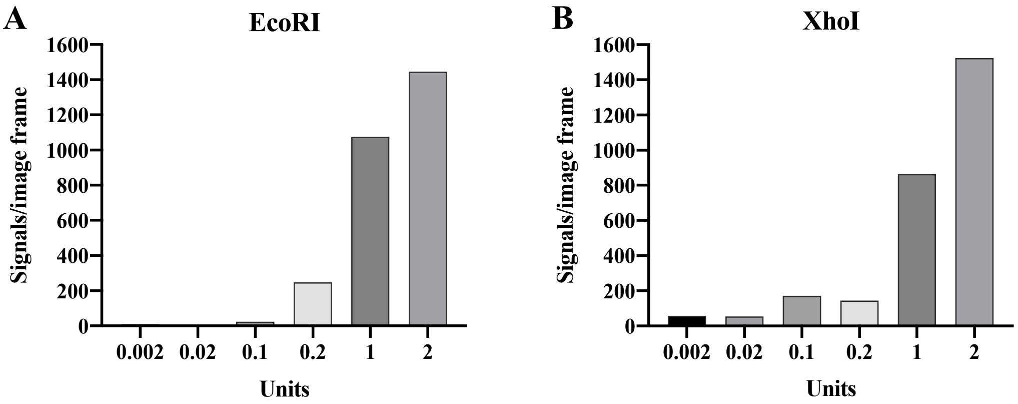

Figure S1. Quantitative detection of purified REs. 0.002 – 2 Units of EcoRI (A) or XhoI (B) were incubated with their target DNA substrate. Data is plotted as the mean of signal/image frame, n = 2.

Supplementary S2

Sequencing of DNA circles

The DNA circles generated using the T4 DNA ligase assisted RE detection method with EcoRI, XhoI and RY13 were supplemented to PCR with phosphorylated primers for the PA and ID sequence in the substrates. The amplified products were subjected to NGS which revealed that the substrates were converted to correct circular products around 80% of the time when using purified REs, as indicated on the schematic depiction of the results. When using the EcoRI expressing RY13 on the EcoRI DNA substrates the correct circular product was found in around 50% of the reads. Using the non-target XhoI substrate, the correct product was found in 20% of the reads. Furthermore, the results indicate that the substrates are digested at multiple sites that are ligated together with the T4 DNA ligase, thus resulting in the generation of false positive signals.

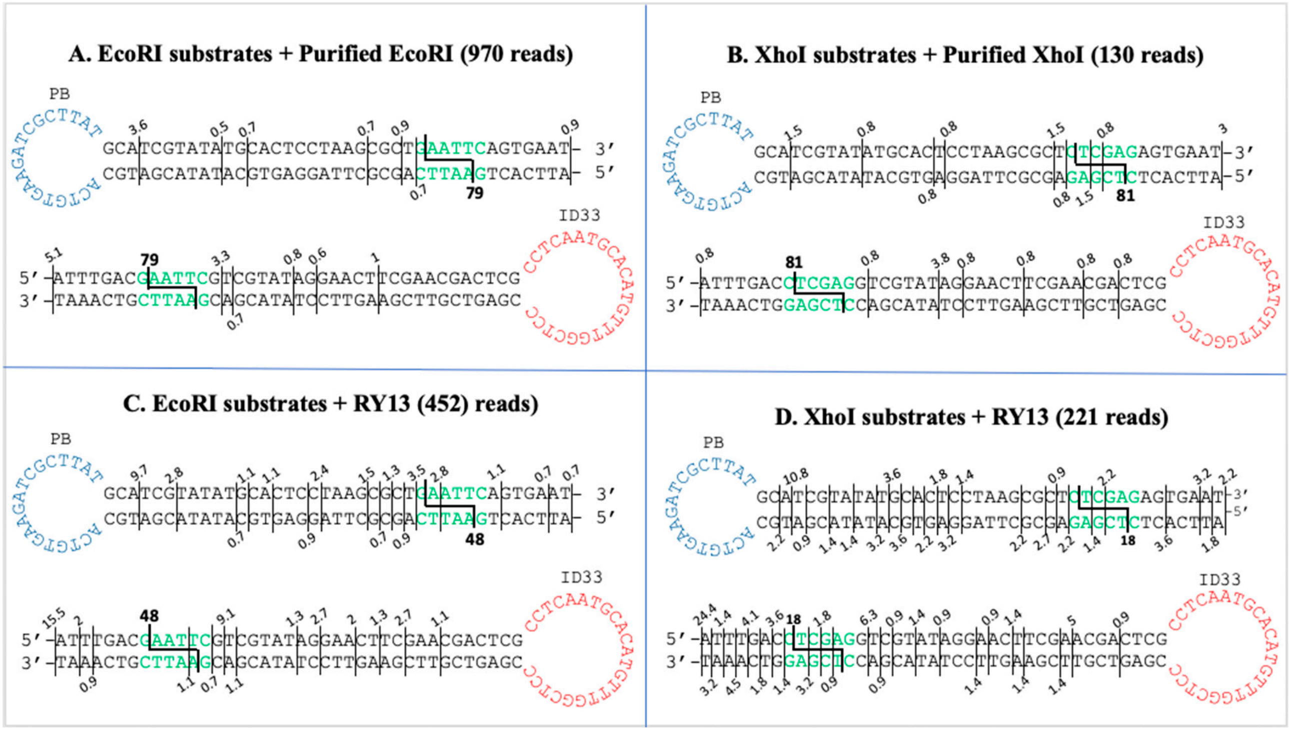

Figure S2. Sequencing of DNA circles. After RE digestion and ligation the circles were subjected to standard PCR with Taq DNA Polymerase with 5′ phosphorylated primers. The PCR products were then sequenced. Sequencing were performed with circles made with EcoRI substrate + purified EcoRI (A), XhoI substrates + purified XhoI (B), EcoRI substrate + RY13 (C) and XhoI substrate + RY13 (D). The number of reads is stated in the figure. Data were analyzed both manually and with UCSC Genome Browser with alignment to the known/expected sequence. The black lines indicate site of cleavage, and the numbers represents the percentage of how many of the reads was cleaved at the particular site.

Supplementary materials and methods

DNA substrates and oligonucleotides

All DNA was purchased Microsynth AG, Switzerland.

- PA PCR primer: 5′-ATAAGCGATCTTCACAGT-3
- ID PCR primer: 5′- GGAGCCAAACATGTGCATTGAGG-3′

Sequencing of DNA circles

The PA and ID PCR primers were phosphorylated using the T4 Polynucleotide Kinase (PNK)(New England Biolabs, Ipswich, MA, USA) in a reaction containing 25 μM primer, 1 unit/μL T4 PNK and 1 mM ATP in 70 mM Tris-HCl pH 7.6, 10 mM $MgCl_2$ and 5 mM DTT for 30 minutes at 37°C. The phosphorylated primers were used to PCR amplify the DNA circles made with RE and T4 DNA ligation with standard Taq DNA polymerase PCR (Invitrogen). The PCR product were then EtOH precipitated. Ion Xpress Barcode Adapters (Thermo Fisher Scientific, Roskilde, Denmark), were ligated to the amplified PCR amplicons. The libraries were quantified using the Ion Library TaqMan Quantitation Kit (Thermo Fisher Scientific, Roskilde, Denmark) and diluted to 50 pM before template preparation and sequencing. Template preparation was performed using the Ion 510 & Ion 520 & Ion 530 Kit—Chef (Thermo Fisher Scientific, Roskilde, Denmark) on the Ion Chef System (Thermo Fisher Scientific, Roskilde, Denmark). Sequencing was performed using the Ion GeneStudio S5 System (Thermo Fisher Scientific, Roskilde, Denmark). Following sequencing, the fastq files were retrieved using the file exporter plugin and the sequences aligned and compared to the substrate sequences using the UCSC Genome Browser and analyzed manually for respective cleavage sites.
